# An independent Monte Carlo–based IMRT QA tool for a 0.35 T MRI‐guided linear accelerator

**DOI:** 10.1002/acm2.13820

**Published:** 2022-11-03

**Authors:** Ahtesham Ullah Khan, Eric A. Simiele, Rajiv Lotey, Larry A. DeWerd, Poonam Yadav

**Affiliations:** ^1^ Department of Medical Physics School of Medicine and Public Health University of Wisconsin‐Madison Madison Wisconsin USA; ^2^ Department of Radiation Oncology Rutgers Cancer Institute of New Jersey, Rutgers Robert Wood Johnson Medical School New Brunswick New Jersey USA; ^3^ ViewRay Inc Oakwood Village Ohio USA; ^4^ Department of Radiation Oncology Northwestern Memorial Hospital Northwestern University Feinberg School of Medicine Chicago Illinois USA

**Keywords:** IMRT QA, log files, Monte Carlo, MR‐guided RT (MRgRT)

## Abstract

**Purpose:**

To develop an independent log file–based intensity‐modulated radiation therapy (IMRT) quality assurance (QA) tool for the 0.35 T magnetic resonance‐linac (MR‐linac) and investigate the ability of various IMRT plan complexity metrics to predict the QA results. Complexity metrics related to tissue heterogeneity were also introduced.

**Methods:**

The tool for particle simulation (TOPAS) Monte Carlo code was utilized with a previously validated linac head model. A cohort of 29 treatment plans was selected for IMRT QA using the developed QA tool and the vendor‐supplied adaptive QA (AQA) tool. For 27 independent patient cases, various IMRT plan complexity metrics were calculated to assess the deliverability of these plans. A correlation between the gamma pass rates (GPRs) from the AQA results and calculated IMRT complexity metrics was determined using the Pearson correlation coefficients. Tissue heterogeneity complexity metrics were calculated based on the gradient of the Hounsfield units.

**Results:**

The median and interquartile range for the TOPAS GPRs (3%/3 mm criteria) were 97.24% and 3.75%, respectively, and were 99.54% and 0.36% for the AQA tool, respectively. The computational time for TOPAS ranged from 4 to 8 h to achieve a statistical uncertainty of <1.5%, whereas the AQA tool had an average calculation time of a few minutes. Of the 23 calculated IMRT plan complexity metrics, the AQA GPRs had correlations with 7 out of 23 of the calculated metrics. Strong correlations (|*r*| > 0.7) were found between the GPRs and the heterogeneity complexity metrics introduced in this work.

**Conclusions:**

An independent MC and log file–based IMRT QA tool was successfully developed and can be clinically deployed for offline QA. The complexity metrics will supplement QA reports and provide information regarding plan complexity.

## 1 INTRODUCTION

Intensity‐modulated radiation therapy (IMRT) is a conformal technique that uses highly modulated radiation beams to target the tumor.^1^ An inverse treatment planning method is typically utilized for IMRT that optimizes beam parameters based on user‐defined dose objectives.^2^ Due to the complexity of these treatment plans, several professional organizations recommend performing pretreatment quality assurance (QA) to assess the plan deliverability.^3^, ^4^, ^5^, ^6^ The QA procedure for IMRT plans typically involves a comparison of the patient‐specific planned dose distribution with the machine‐delivered dose distribution that can either be measured or computationally calculated using machine log files.^7^, ^8^, ^9^, ^10^, ^11^ Quantitative comparison of the dose distributions is usually performed with gamma analysis.^12^ Log files are generated by most linear accelerators (linacs) following delivery and contain plan delivery information, such as multileaf collimator (MLC) positions, gantry angles, monitor units (MUs), and dose rate. There is an ongoing debate on the use of measurement‐based IMRT QA versus log file–based IMRT QA.^13^, ^14^, ^15^, ^16^ A few advantages of the log file–based QA procedure include comparison of 3D dose distribution using the patient's CT scan instead of homogeneous phantoms, post treatment verification of the dose distribution, and its ability to be highly automated.^10^, ^13^ However, the reliability of the log files to accurately capture the machine parameters has been questioned in the literature.^17^ Therefore, a rigorous QA program assessing the accuracy of the information depicted in the log files is desired if log file–based IMRT QA is to be implemented.

With the increased use of magnetic resonance imaging (MRI) guidance in radiation therapy, a routine visualization of patient anatomy has enabled adaptive radiotherapy (ART).^18^, ^19^, ^20^, ^21^, ^22^, ^23^ When utilizing ART, the initially planned treatment may be revised multiple times throughout the treatment course depending on changes in the patient's anatomy.^24^, ^25^ Recommendations for performing QA for the adapted plans are not widely discussed. Online ART QA is especially challenging because the patient is on the couch during the adaptation process, and the usage of measurement‐based QA is difficult.^26^ Conventional dose calculation methods such as convolution superposition can be employed along with the log files to perform ART QA when the radiation beams are not subjected to strong magnetic fields with the computational time of several minutes. However, the use of Monte Carlo (MC) simulations for dose calculation is desired for MR‐guided treatment machines to accurately assess the dose distributions in the presence of a strong magnetic field.^27^ Previously, a fast MC dose calculation system was developed that allows clinically–feasibly dose calculation times for a ^60^Co MRI‐guided teletherapy machine.^27^, ^28^ GPU‐accelerated MC treatment planning systems (TPS) for MR‐linacs have since been introduced.^29^ However, a large majority of these fast MC codes are either proprietary in nature or lack extensive evaluation of their transport physics. Additionally, a dose calculation platform, independent of the vendor‐provided TPS, is desired.^14^, ^30^ Although common general‐purpose MC codes, such as EGSnrc, Geant4, PENELOPE, and MCNP6, have been assessed using electrons under the influence of strong magnetic fields, the high computational time needed to calculate absorbed dose for a typical IMRT treatment plan using these codes limits their use in creating clinical QA tools.^31^ Nevertheless, variance reduction techniques can be used to substantially decrease computational time.^32^ As these general‐purpose codes have been widely evaluated and trusted in terms of accuracy, it is important to compare the fast MC dose calculation codes with the general‐purpose codes to ensure that accuracy is retained when a fast solution is implemented.

For the commercially available 0.35 T MR‐linac system, a vendor‐supplied adaptive QA (AQA) tool that implements log file–based IMRT QA is currently available.^33^ Using the plan parameter information from the delivery log file, the AQA tool is used to calculate absorbed dose distributions in a virtual phantom composed of material extracted from the patient's CT scan. The AQA tool considers the impact of the external magnetic field on the electron transport. The TPS‐planned and AQA‐calculated dose distributions are then compared using a 3D gamma analysis, and a gamma pass rate (GPR) is calculated.^12^ The AQA tool utilizes a phase space file generated by the TPS beam model above the MLC plane and uses an independent fast MC code to transport the scored particles through the MLC banks and the patient geometry. Although partially independent and proprietary in nature, the AQA tool allows calculation‐based IMRT QA for ART treatments with a computational time of few minutes. However, there is still a need to build a vendor and TPS‐independent IMRT QA tool that utilizes validated and well‐known MC transport algorithms and interaction data. This work aimed to develop such a tool using log files and a previously validated MC linac head model of a 0.35 T MR‐linac.^34^ Log file–based IMRT QA was performed for 10 patients undergoing ART (*N* = 29 treatment plans including adapted plans). The IMRT QA results from the developed tool were compared with the AQA tool for the same patient cohort.

In addition to independent secondary dose calculation checks, IMRT complexity metrics can further aid in the evaluation of accurate plan delivery and can be used to optimize the IMRT QA program to only QA plans that are predicted to be more complex.^35^ Over the past decades, a large number of complexity metrics have been proposed based on various plan parameters, such as dose rate, MLC aperture shape and size, MLC leaf speed and travel, gantry speed and angles, and delivered MUs. The ideal plan complexity metric would be able to accurately predict the deliverability of any given plan. However, that is rarely the case and, therefore, it is best to utilize multiple complexity metrics for plan assessment purposes. Desai et al. previously correlated various IMRT plan complexity metrics with GPRs measured using commercial diode array–based homogeneous phantoms for the 0.35 T MR‐linac.^36^ It was found that the correlation of complexity metrics with GPRs was detector‐dependent. Therefore, it is important to investigate and identify the complexity metrics that best predict the QA results for the AQA tool.

The presence of a strong external magnetic field induces an electron return effect (ERE), which complicates the dosimetry of MR‐guided radiotherapy.^37^ In our previous work comparing the TPS dose distributions with the Geant4 MC code in heterogeneous phantoms, the largest disagreement was noted to be at the interfaces consisting of high‐ and low‐density media.^34^ Hence, the presence of such interfaces in the patient's anatomy is directly related to the IMRT QA results and plan complexity. In addition to the previously published plan complexity metrics, two new complexity metrics were introduced in this work to quantify the tissue heterogeneity.

## 2 MATERIALS AND METHODS

All MC simulations in this work were run as 100 concurrent‐independent simulation jobs (100 cores) on the University of Wisconsin‐Madison Center of High Throughput Computing (CHTC) cluster.

### 2.1 MRIdian 0.35 T MR‐linac

The MR‐linac considered in this work combines a 6 MV flattening filter‐free photon beam with a 0.35 T magnetic field.^33^ The direction of the magnetic field is in the inferior‐to‐superior direction, which is orthogonal to the direction of both the photon beam propagation and MLC motion. The photon beam is collimated by dual‐stacked and dual‐focused MLC banks with a leaf width of 0.83 cm at isocenter.^38^ The top and bottom stacks are offset by half a leaf width to enhance the collimation resolution (0.415 cm in the inline direction) and to mitigate the tongue and groove effect. All MLC leaves have a height of 5.5 cm and are composed of a 17.7 g/cm^3^ non‐ferromagnetic tungsten alloy with the top stack housing 68 dynamic leaves and the bottom stack containing 70 dynamic leaves. The source‐to‐axial distance for the linac is 90 cm with a nominal dose rate of 600 MU/min. The delivery techniques used by this linac include 3D conformal and step‐and‐shoot IMRT.

### 2.2 Comparison of the log file–based QA tool (AQA) with TOPAS MC code

The MC model of the 0.35 T MR‐linac has been previously benchmarked for the specific MR‐linac under investigation.^34^ Detailed modeling of the linac's head was performed in the Geant4 MC code and was validated using experimental data such as percent depth–dose (PDD) curves, lateral beam profiles, and output factors.^39^ In this work, the tool for particle simulation (TOPAS v3.7.0) MC code was utilized, and the Geant4 MR‐linac head model was imported as a TOPAS extension.^40^ There are no differences between the two codes as TOPAS is a Geant4 wrapper. The physics list used in this work was the same as previously described.^34^ The production thresholds were set to 1 mm in the patient and the linac target region and were overridden to 1 cm in the MLC region. To validate the electromagnetic physics list and to ensure a high accuracy of the multiple Coulomb scattering algorithm when exposed to a magnetic field, a Fano cavity test was implemented in accordance to the work of Simiele and DeWerd, and it was ensured that the employed physics list passes the test within a 0.5% criteria.^41^ A phase space file was generated above the MLC banks, and directional bremsstrahlung splitting (DBS) was utilized with a splitting number of 1000 to increase the computational speed of the simulations.^42^

For any given patient plan, the IMRT QA tool used the same phase space file for each segment, and particles were tracked through both MLC banks and MRI coils before they reached the patient. The patient CT was imported into TOPAS, and the Hounsfield units (HU) were converted to material composition and mass density using the Schneider method.^43^ A virtual voxelized phantom was created to encompass the entire patient CT volume with a voxel size of 2 × 3 × 2 mm^3^ with 3 mm in the axial direction (superior–inferior), which is parallel to the magnetic field direction. Shifts were applied to the CT volume to position the MR‐linac isocenter at the planned location. Using the time features in the TOPAS MC code, the gantry angle and MLC leaf positions were dynamically updated for each segment. A custom dose to medium scorer was implemented to weight the scored absorbed dose by the segment MUs. In order to further speed up the simulations, geometrical importance sampling was used with the CT volume assigned to an importance value of 20 relative to the remaining geometry.^44^ Five million histories per segment were run to achieve a statistical uncertainty of <1.5% for the composite‐absorbed dose.

A 3D median filter (3 × 3 × 3) was applied to the TOPAS‐simulated dose distribution to reduce random noise. The TPS dose distribution was resized to match the TOPAS voxel size. Following the simulations, the relative TOPAS calculated and TPS planned 3D absorbed dose distributions were compared using a global gamma analysis, and GPRs were calculated using a 3%/3 mm criteria for a dose threshold of 10% of the maximum dose.^12^ For the same patient plan, the MRIdian AQA tool was used to calculate the GPR using a 3%/3 mm criteria with a 10% dose threshold. Ideally, the comparison between the calculated 3D dose distribution for TOPAS and AQA tools would be performed directly using a GPR. However, 3D dose distribution is currently not obtainable from the AQA software.

A total of 29 IMRT plans (belonging to ten patients) were retrospectively selected for log file–based IMRT QA. The stereotactic body radiation therapy technique was used for all cases. Table 1 classifies the cases and treatment plans according to their respective anatomical categories. Most of these cases belonged to the tumors in the region where target motion is substantial and can be visualized using MRI guidance. For multiple plans, more than one planning target volumes were treated. The CT imaging volume and planned dose distribution for these patients had a slice thickness of 3 mm and pixel size of 1.5 mm. The log files generated following delivery QA were exported for each treatment plan. A MATLAB v2020a (Natick, MA) script was written to create TOPAS input files from individual log files containing information, such as the gantry angle, MUs, and MLC positions for each segment in each IMRT plan.

**TABLE 1:** Distribution of the investigated plans over anatomical sites for validation of adaptive quality assurance (AQA) with tool for particle simulation (TOPAS)

Anatomical site	Number of cases	Number of plans
Pancreas	3	8
Liver	2	3
Lung	1	4
Kidney	1	2
Abdomen	1	5
Pelvis	1	4
Prostate	1	3

### 2.3 Correlation of AQA results with plan complexity metrics

**FIGURE 1:**
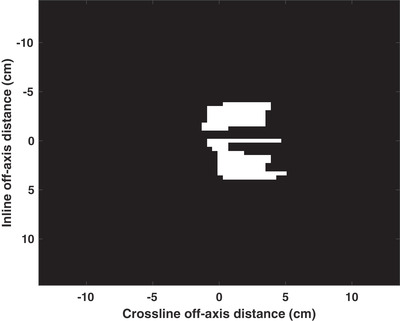
Example of a composite binary image representing the segment aperture for a lung stereotactic body radiation therapy (SBRT) treatment plan

IMRT plan complexity metrics were calculated using the plan parameters in the log file. A total of 27 independent patient cases were considered with the anatomical sites listed in Table 2. For each patient, a single adapted plan was selected arbitrarily. Theoretically, the agreement between the patient‐specific planned and measured/calculated dose distribution would decrease with increasing IMRT plan complexity. However, quantifying the complexity of a given plan is not a trivial task. Previously, various plan complexity metrics have been proposed to predict the agreement between the planned and measured/calculated dose distribution.^35^ In this work, the first 21 of these metrics, described in Table 3, were utilized to assess the complexity of the treatment plans using the planning log files. Two additional complexity metrics were also introduced. For each segment in each plan, MLC positions for both the top and bottom stacks were extracted from the log files and converted into binary images of 0.01 × 0.415 cm^2^ resolution. As shown in Figure 1, a composite binary image was created for each segment by multiplying the binary images for the top and bottom MLC stacks. All plan complexity metrics were calculated using the composite binary images and the associated MUs and gantry angles. Two complexity metrics, tissue heterogeneity index (THI) and dose‐weighted tissue heterogeneity index (DWTHI), were introduced to quantify the patient tissue heterogeneity and the presence of regions where ERE can induce a disagreement between the TPS and AQA dose distribution:

$$\begin{array}{ccc} & & \mathrm{THI}=\\ & & \sum_{D>10\%}\sqrt{{\left(\frac{\partial \mathrm{HU}\left(x,y,z\right)}{\partial x}\right)}^{2}+{\left(\frac{\partial \mathrm{HU}\left(x,y,z\right)}{\partial y}\right)}^{2}+{\left(\frac{\partial \mathrm{HU}\left(x,y,z\right)}{\partial z}\right)}^{2}} \end{array}$$

$$\begin{array}{ccc} \mathrm{DWTHI} & = & \sum_{D>10\%}D_{\mathrm{rel}}\left(x,y,z\right)\\ & & \sqrt{{\left(\frac{\partial \mathrm{HU}\left(x,y,z\right)}{\partial x}\right)}^{2}+{\left(\frac{\partial \mathrm{HU}\left(x,y,z\right)}{\partial y}\right)}^{2}+{\left(\frac{\partial \mathrm{HU}\left(x,y,z\right)}{\partial z}\right)}^{2}} \end{array}$$

where HU(*x*,*y*,*z*) is the Hounsfield units, and *D*_rel_ is the relative absorbed dose at a given location. The THI and DWTHI values were calculated for voxels receiving relative absorbed dose of over 10% of the maximum dose.

**TABLE 2:** Anatomical sites of the patient cases for the intensity‐modulated radiation therapy (IMRT) plan complexity calculations

Anatomical site	Number of cases
Pancreas	5
Lung	5
Liver	4
Kidney	3
Adrenal	3
Cardiac	2
Stomach	1
Prostate	1
Abdomen	1
Spleen	1
Pelvis	1

**TABLE 3:** Summary of the plan complexity metrics used in this work

Complexity metric	Description
Cumulative area	Total area of the apertures weighted by segment MUs
Cumulative perimeter	Total perimeter of the apertures weighted by segment MUs
Perimeter‐to‐area ratio	Ratio of the cumulative perimeter to cumulative area
Edge metric^45^	Total perimeter of the aperture excluding the contributions from the leaf tips
Number of active MLC pairs	Total number of MLC pairs creating an opening
Average leaf pair opening (ALPO)^46^	Mean leaf opening distance weighted by segment MUs
Leaf sequence variability (LSV)^47^	Variability in the segment aperture shape based on variation in the adjacent MLC leaf positions
Aperture area variability (AAV)^47^	Variation in the segment aperture area relative to the maximum aperture area over all the segments
Modulation complexity score (MCS)^47^	Product of LSV and AAV
Aperture irregularity^48^	Non‐circularity of the aperture
Unique opening index	Number of noncontiguous apertures in a plan
Cross‐axis score	Fraction of MLC leaves that cross over the central axis
Small aperture score 5 mm	Fraction of MLC pairs with an opening less than 5 mm
Small aperture score 10 mm	Fraction of MLC pairs with an opening less than 10 mm
Small aperture score 20 mm	Fraction of MLC pairs with an opening less than 20 mm
Mean aperture displacement (MAD)^49^	Average displacement between the midway point between each pair of leaves and the midline
Leaf travel index (LTI)^50^	Average leaf travel over all segments normalized to 100 cm
Leaf travel index modulation complexity score (LTIMCS)^50^	Product of (1‐LTI/10) and MCS
Number of segments	Total number of segments in a plan
MUs per segment	Average relative MUs per segment
Total MUs	Cumulative MUs
Tissue heterogeneity index (THI)	Sum of HU spatial gradient above a dose threshold
Dose‐weighted tissue heterogeneity index (DWTHI)	Sum of HU spatial gradient weighted by the computed relative absorbed dose

Abbreviations: MLC, multileaf collimator; MU, monitor unit.

For each complexity metric, the Pearson correlation coefficients were calculated for the AQA‐calculated GPRs. Only statistically significant correlations were considered in this study.

## 3 RESULTS

### 3.1 Comparison of the log file–based QA tool (AQA) with TOPAS MC code

**FIGURE 2:**
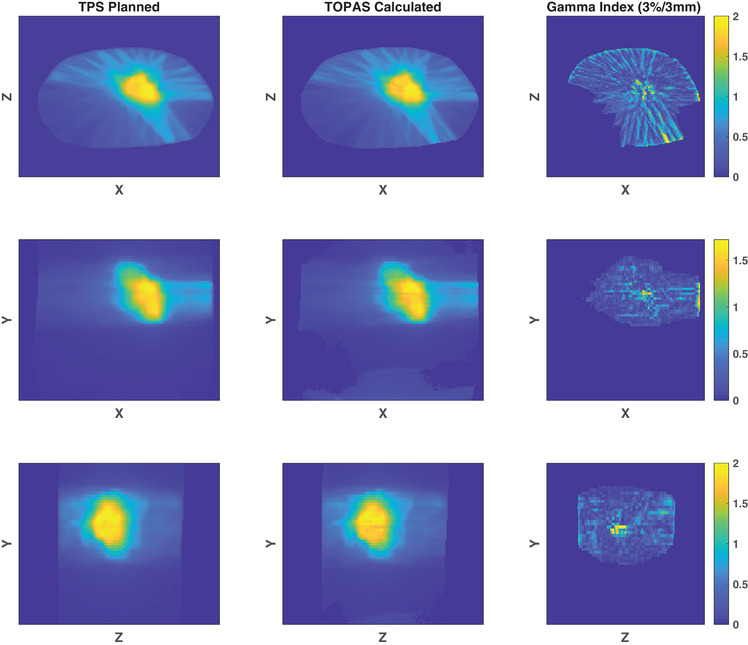
Planned (first column) and delivered (second column) dose distributions for a pancreas treatment plan along with the gamma index distribution (third column) for a single axial (*xz*), coronal (*xy*), and sagittal (*zy*) slice. The gamma indices were calculated using a 3%/3 mm criterion.

Figure 2 shows an example of a TOPAS‐reconstructed 3D absorbed dose distribution for a pancreas treatment plan. A computational run time of 5 h was required to achieve a mean statistical uncertainty of 0.24% with a one standard deviation of 0.5%. The GPR between the TPS‐planned and TOPAS‐calculated dose distributions was 97.6% (3%/3 mm), and the average gamma index was determined to be 0.29. The largest disagreement, not unexpectedly, was at the interfaces of the soft tissue and air cavities in the abdomen near the pancreas.

**FIGURE 3:**
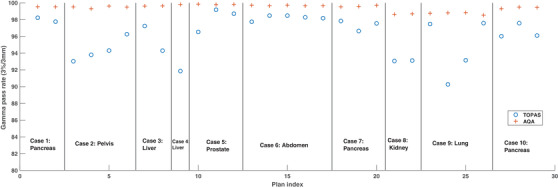
A comparison of gamma pass rates (GPRs) between the tool for particle simulation (TOPAS) and the adaptive quality assurance (AQA) tools using a 3%/3 mm criteria and a 10% threshold for all cases, including the adapted treatment plans. The GPRs for each patient are separated by a vertical line

For the cohort of patient cases selected for this investigation, the mean number of segments per plan was 77, and the mean time required for a single segment was 4 min. The mean time required to simulate a treatment plan was 5 h. Figure 3 shows the GPRs calculated for all patient cases using the AQA tool and the TOPAS MC code using a 3%/3 mm criteria. The AQA GPRs were observed to be significantly higher than the TOPAS GPRs with the mean GPR being 99.4% for the AQA tool and 96.2% for the TOPAS tool. Regardless, all TOPAS GPRs were greater than 90%. The lower TOPAS GPRs were observed for the lung‐and‐liver plans, which typically have large tissue heterogeneities. The adapted plans displayed a much greater variability for the TOPAS QA tool as compared to the AQA tool. The maximum range in the GPRs for the adapted plans was calculated to be 0.31% for the AQA tool and 3.23% for the TOPAS tool. These results demonstrate that the adaptations made to the original treatment plans when using ART were deemed minor by the AQA tool but can be considered significant when using the TOPAS QA tool. The changes in the patient anatomy over the course of the treatment, such as changes in the location and sizes of the air cavities, were attributed to be the major cause of the variability in the GPRs. Therefore, IMRT QA is important to perform when employing ART.

### 3.2 Correlation of AQA results with plan complexity metrics

The mean and relative standard deviation values for all plan complexity metrics are shown in Table 4. The selected treatment plans displayed a large variation in the complexity metrics with the smallest percent standard deviation being 9.87% for the leaf sequence variability (LSV) metric and the largest percent standard deviation being 362.92% for the MUs per segment metric. A large variation in the THI and DWTHI metrics was found due to a wide variety of anatomical sites chosen for this study. The average THI and DWTHI values for the lung plans were found to be 51% and 66% greater than the total average THI and DWTHI, respectively.

**TABLE 4:** Mean and associated percent standard deviation for the complexity metrics considered in this work across all patients, plans, and treatment sites

Complexity metric	Mean value	Percent st. dev. (%)
Cumulative area (cm^2^)	20.57	74.98
Cumulative perimeter (cm)	27.76	50.63
Perimeter‐to‐area ratio (1/cm)	1.03	28.26
Edge metric (cm)	1.59	31.20
Number of active MLC pairs	579.2	77.82
ALPO (cm)	6.46	34.30
LSV	0.72	9.87
AAV	1.04	26.37
MCS	0.78	29.90
Aperture irregularity	3.86	46.37
Unique opening index	133.3	66.00
Cross‐axis score	0.32	32.99
Small aperture score 5 mm	0.02	164.74
Small aperture score 10 mm	0.09	98.89
Small aperture score 20 mm	0.26	59.94
MAD (cm)	2.75	65.28
LTI	1.40	87.38
LTIMCS	0.52	40.02
Number of segments	75.85	39.72
MUs per segment	4.34	362.92
Total MUs	3579.86	50.46
THI (HU/mm)	10 213 755	110.00
DWTHI (HU/mm)	3769 409	131.39

Abbreviations: AAV, aperture area variability; ALPO, average leaf pair opening; DWTHI, dose‐weighted tissue heterogeneity index; LSV, leaf sequence variability; LTI, leaf travel index; LTIMCS, leaf travel index modulation complexity score; MAD, mean aperture displacement; MCS, modulation complexity score; MLC, multileaf collimator; THI, tissue heterogeneity index.

The correlation data between the GPRs and various plan complexity metrics for the AQA data are shown in Table 5. Statistically significant correlations were found between the GPRs and 7 out of 23 of the complexity metrics for the AQA tool. The negative correlations for the cumulative area, cumulative perimeter, and average leaf pair opening (ALPO) metrics were unexpected because highly complex plans usually contain smaller apertures, and small fields typically lead to worse GPRs. These correlations were also noted to be statistically strong making these metrics good predictors of the GPR. The correlations for the LSV and aperture irregularity were found to be moderate. Aperture irregularity can potentially be used for GPR assessment if a moderate correlation can be considered statistically sufficient. A negative correlation between LSV and AQA GPRs was unexpected. The LSV metric characterizes the shape of the segment apertures where rectangular apertures result in an LSV of 1 and more irregular apertures resulting in LSV values less than unity. Therefore, a positive correlation between LSV and GPR was expected because rectangular fields are considered less complex. A strong negative correlation was observed between the THI/DWTHI and AQA GPRs, which was hypothesized because greater heterogeneity enhances ERE compared to homogenous media leading to lower GPRs. Both THI and DWTHI were equally found to be good predictors of AQA GPRs.

**TABLE 5:** Correlation coefficients and associated *p*‐values for the adaptive quality assurance (AQA) 3%/3 mm gamma pass rates (GPRs) and several plan complexity metrics

Complexity metric	AQA Pearson *r* value	AQA *p*‐value
Cumulative area (cm^2^)	**−0.81**	**0.00**
Cumulative perimeter (cm)	**−0.76**	**0.00**
Perimeter to area ratio (1/cm)	0.23	0.25
Edge metric (cm)	0.41	0.03
Number of active MLC pairs	−0.49	0.01
ALPO (cm)	**−0.75**	**0.00**
LSV	**−0.53**	**0.00**
AAV	−0.22	0.27
MCS	−0.36	0.06
Aperture irregularity	**−0.51**	**0.01**
Unique opening index	−0.30	0.13
Cross‐axis score	0.17	0.40
Small aperture score 5 mm	0.18	0.36
Small aperture score 10 mm	0.19	0.34
Small aperture score 20 mm	0.26	0.20
MAD (cm)	−0.10	0.61
LTI (cm)	0.12	0.54
LTIMCS (cm)	0.12	0.54
Number of segments	−0.22	0.26
MUs per segment	−0.28	0.15
Total MUs	0.13	0.50
THI (HU/mm)	**−0.73**	**0.00**
DWTHI (Gy HU/mm)	**−0.70**	**0.00**

*Note*: The statistically significant correlations are bolded.

Abbreviations: AAV, aperture area variability; ALPO, average leaf pair opening; DWTHI, dose‐weighted tissue heterogeneity index; LSV, leaf sequence variability; LTI, leaf travel index; LTIMCS, leaf travel index modulation complexity score; MAD, mean aperture displacement; MCS, modulation complexity score; MLC, multileaf collimator; MU, monitor unit; THI, tissue heterogeneity index.

**FIGURE 4:**
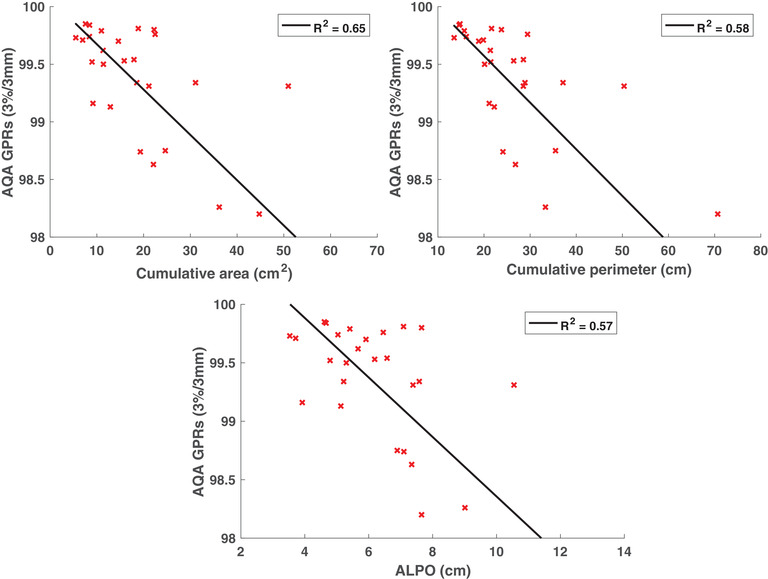
Adaptive quality assurance (AQA) gamma pass rates (GPRs), evaluated with a 3%/3 mm criteria, plotted against complexity metrics related to multileaf collimator (MLC) field size and shape, and considered to be good predictors of GPRs

**FIGURE 5:**
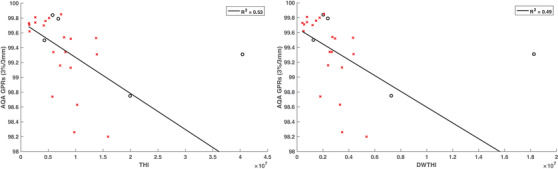
Adaptive quality assurance (AQA) gamma pass rates (GPRs), evaluated with a 3%/3 mm criteria, plotted against various complexity metrics related to tissue heterogeneity, and considered to be good predictors of GPRs. The circular data points show the results for the lung cases

Figures 4 and 5 show the AQA GPRs against relevant IMRT complexity metrics that were observed be good predictors of plan complexity. Although statistically significant correlations were found in this study between GPRs and multiple IMRT complexity metrics, the presence of several outliers weakens the reliability of these metrics. Therefore, the complexity of a given plan should not be solely assessed using a single metric.

## 4 DISCUSSION

To our knowledge, this study reports the first independent log file–based IMRT QA tool for the 0.35 T MR‐linac. A well‐studied and open‐source MC code was utilized, with a physics list validated using a Fano cavity test and linac beam model previously validated using experimental data, to calculate absorbed dose in a voxelized patient CT imaging set using the plan information extracted from the delivery log files. The TOPAS GPRs were compared to the vendor‐supplied log file–based AQA tool. The AQA tool showed much higher GPRs for all evaluated plans compared to TOPAS, which can be partially attributed to the semi‐independent nature of the AQA tool. The phase space file utilized by the MC‐based AQA software used the beam model from the TPS creating a correlation between the TPS and AQA photon beam. Additionally, any differences in the CT number to material convertor, cross section data, and linac head and MLC modeling between the two codes can further lead to differences in the calculated absorbed dose. Although the TOPAS MC code required several hours to perform IMRT QA, the AQA tool was able to calculate absorbed dose in a few minutes limiting the use of the TOPAS QA tool for online AQA. Regardless, the developed IMRT QA tool can be utilized as a research tool and act as a secondary independent check for the TPS and the AQA tool in an offline setting. It is of note that the developed TOPAS tool can be utilized for MRIdian machines other than the specific machine at our clinic as the dosimetric properties of the photon beam were previously found similar between different linacs.^51^

Previously, Friedel et al. modeled the linac head of the 1.5 T MR‐linac in the EGSnrc MC code and calculated absorbed dose to a patient's CT dataset for a prostate cancer case.^52^ A computational time of ∼9 h was reported, and a GPR of 99.83% was calculated using a 3%/3 mm criteria. The mean TOPAS GPR calculated in this work for the prostate case was 98.14%, which is similar to the results shown in the work of Friedel et al., and the computational time was also observed to be similar.

Recently, Nachbar et al. created a fast MC‐based IMRT QA tool for the 1.5 T MR‐linac while ignoring the effect of the magnetic field on the absorbed dose distributions.^53^ Their work introduced multiple ways of performing adaptive IMRT QA with both a precision offline criterion (POC) method and a fast online method. Their reported GPRs varied largely based on the anatomical site with breast and head‐and‐neck (H&N) GPRs being much lower than other sites. It is worth noting that lung treatment plans were not included in their study where the influence of the magnetic field can be quite large. Overall, their calculated POC GPRs ranged from 82% to 99.5% with the calculation time of 6–12 min. Therefore, it is important to consider the effect of the magnetic field on absorbed dose distribution when significant tissue heterogeneity is encountered.

Li et al. developed an extension for a previously existing GPU‐based MC code to allow independent IMRT dose verification for the 1.5 T MR‐linac.^54^ To achieve a statistical uncertainty of ∼1%, a computational time of <40 s was reported in their work. GPRs (3%/2 mm criteria) of 93%–98% were calculated in their work for treatment plans belonging to various anatomical sites, including lung and H&N, which is close to the results observed in this study.

Although several studies have previously employed conventional dose calculation algorithms such as convolution superposition in order to perform independent IMRT QA for MR‐linacs, any treatment plans containing large tissue heterogeneities yielded GPRs of <90%.^55^, ^56^ The IMRT plan complexity metrics introduced in this work, such as THI and DWTHI, aim to quantify the tissue heterogeneity and can be used to determine the appropriate IMRT QA tool to use for a given plan. The THI and DWTHI metrics were found to be strongly correlated with the AQA GPRs. A major limitation of this study is that only 27 patients were included for the IMRT complexity metrics calculations. In the future, a larger TOPAS GPR dataset will be utilized with diverse anatomical sites to study the impact of tissue heterogeneity on IMRT QA results.

For the AQA tool, several complexity metrics were found to be correlated with the GPRs. However, several of these correlations were unexpected or weak. A nonlinear relationship between the GPRs and plan complexity metric is possible but was not explored in this work, which is a limitation of this work. In our previous study simulating phantoms with heterogeneous media, the ERE was found to be dependent on the field size.^34^ The PDD curves for the 0.35 and 0 T magnetic field strengths in a water–lung–water phantom were found to be closer to each other for the smaller field size. Although small fields are considered more complex in the context of dosimetry, the possible ERE dependence on the field size can partially explain the strong negative correlations found between the AQA GPR and the area, perimeter, and ALPO metrics.

The work of Desai et al. aimed to correlate the GPRs from ArcCHECK‐MR and Delta4 Phantom+ MR with many of the complexity metrics included in this work.^36^ The ArcCHECK GPRs were observed to be independent of the plan complexity metrics except two moderate correlations were found. However, the Delta4 phantom GPRs, like the AQA GPRs, displayed strong correlations with various complexity metrics. Similar to this work, negative correlations were observed for the area, perimeter, LSV, and aperture irregularity metrics. However, unlike this work, a statistically significant correlation was not found between the GPRs and the ALPO metric. The strongest correlation was reported for the plan irregularity metric in their work, which only had a moderate correlation with the AQA GPRs in this study. Unlike their work, the results shown in this work were not categorized into different anatomical sites due to the limited number of available plans and can be considered a limitation of this study.

## 5 CONCLUSIONS

An independent MC‐ and log file–based IMRT QA tool was successfully developed in this work for the 0.35 T MR‐linac and was compared to the vendor‐provided AQA tool. The developed QA tool was determined to be not clinically feasible for online ART, however, can be clinically employed for offline IMRT QA. The average GPR between the TPS‐planned and TOPAS‐calculated absorbed dose distributions was calculated to be 96.2% ± 2.4% for 10 patients with a total of 29 treatment plans. The AQA tool showed a much higher mean GPR of 99.4% ± 0.4% compared to the TOPAS QA tool. A few correlations between the AQA GPRs with various IMRT plan complexity metrics were found, which can be useful when optimizing any IMRT QA program to only QA a fraction of the total treatment plans.

## AUTHOR CONTRIBUTIONS

Ahtesham Ullah Khan, Eric A. Simiele, Rajiv Lotey, Larry A. DeWerd, and Poonam Yadav, all contributed to the preparation of the manuscript. Poonam Yadav provided the scientific direction. Poonam Yadav and Larry A. DeWerd supervised the work. Ahtesham Ullah Khan performed the experiments and gathered data. All authors analyzed the data and participated in the discussion.

## CONFLICT OF INTEREST

Rajiv Lotey is an employee of ViewRay, Inc.

## Data Availability

The data that support the findings of this study are available from the corresponding author upon reasonable request.
